# Nonlinear dynamics and mechanisms of digital inclusive finance on county-level public services in China: Threshold and mediation analyses

**DOI:** 10.1371/journal.pone.0327771

**Published:** 2025-07-17

**Authors:** Xin Huang, Feng Lan

**Affiliations:** 1 School of Management, Xi’an University of Architecture and Technology, Xi’an, China; 2 Research Center of Green Development and Mechanism Innovation of Real Estate Industry in Shaanxi Province, Xi’an University of Architecture and Technology, Xi’an, China; PLoS ONE, UNITED STATES OF AMERICA

## Abstract

Counties, as fundamental administrative units in China, play a pivotal role in advancing digital inclusive finance and improving public services. Despite their significance in bridging regional disparities and promoting grassroots development, research focusing specifically on counties remains insufficient. This article aims to explore the relationship between digital inclusive finance and county-level public service quality by constructing a comprehensive evaluation system. Selecting data from 1445 counties across three regions of China between 2008 and 2022, public service quality across five dimensions—education, healthcare, ecology, municipal, and transportation facilities—is assessed through the entropy weighting method. Empirical analyses using threshold, intermediary, and moderation effect models reveal that digital inclusive finance enhances livelihood-related services but negatively affects infrastructure-related services. A “U-shaped” nonlinear trend is identified in the impact of credit depth, with the development of the primary industry mitigating negative effects. Mediation analysis shows that digital inclusive finance can improve public services through two pathways: boosting the tertiary industry and increasing financial institution loans. This article offers theoretical and practical guidance for integrating digital inclusive finance to enhance the quality and equity of county-level public services.

## 1. Introduction

County-level public services in China refer to the essential services provided by the government—such as education, healthcare, social security, and infrastructure—that are crucial for meeting residents’ basic needs and promoting social equity (Wang and Chi, 2018 [1]; Wang et al., 2024 [2]). In China’s administrative system, counties serve as key grassroots units for implementing national policies, delivering services to both urban and rural populations. However, counties face a significant, real-world dilemma: uneven resource distribution, especially in education and healthcare, combined with fiscal constraints and limited service capacity (Jia and Cheng, 2025 [3]; Wang and Wang, 2023 [4]). These challenges not only impede effective public service delivery in economically disadvantaged counties but also exacerbate socio-economic disparities. Addressing this dilemma is essential, as it directly affects millions of residents and hinders balanced regional development.

To mitigate these challenges, this study examines the role of digital inclusive finance—a mechanism that uses digital technologies to offer affordable and accessible financial services to underserved groups (Chen and Zhao, 2021 [5]). This research is particularly important because it addresses a critical gap in the literature by focusing on county-level dynamics, an area where financial inclusion has been underexplored yet is essential for reducing regional disparities and improving public service delivery. Furthermore, the innovative nature of digital inclusive finance, characterized by its integration of advanced technologies like blockchain and artificial intelligence, offers novel insights that can significantly enhance both theoretical understanding and practical policymaking, making this study highly relevant for both academia and real-world applications.

By providing digital payments, online lending, and microinsurance, digital inclusive finance can reduce credit barriers and channel financial resources to areas in need (Hasan et al., 2022 [6]). Beyond its general role, digital inclusive finance significantly enhances specific public service sectors such as education, healthcare, and culture. Foundational insights provided by Kanungo and Gupta (2021) [7] demonstrate that financial inclusion improves access to these essential services, while Ozili (2021) [8] underscores global evidence of its impact on service quality in low-income regions. Mhlanga (2023) [9] further emphasizes that innovations like blockchain can effectively redirect resources to vulnerable sectors, and Tran and Le (2021) [10] highlight its poverty-reduction effects and capacity to stimulate social demand. These functions suggest that digital inclusive finance holds promise as an innovative solution to the pressing problems faced by county-level public services.

Despite the recognized benefits of digital inclusive finance, its application in county-level regions remains underexplored. Compared to urban centers, counties often suffer from underdeveloped financial infrastructure, limited access to digital platforms, and weaker institutional support. For instance, Martinez and Krauss (2015) [11] discuss how geographic and institutional barriers impede financial inclusion in rural areas, a challenge mirrored in county-level regions. Moreover, traditional financial models are proving increasingly inadequate, calling for research that uncovers how digital finance can overcome these obstacles (Günther, 2017) [12]. This research gap underscores the importance of this study: to rigorously analyze how digital inclusive finance affects public service quality and to uncover the mechanisms behind its impact.

The guiding research questions of this study are explicitly centered on understanding the effects of digital inclusive finance on public service provision at the county level. Specifically, this study focus on: (i) What are the direct and indirect effects of digital inclusive finance on public service quality? (ii) Through which mechanisms—such as financial resource reallocation, industrial upgrading, and improvements in the lending environment—does digital inclusive finance enhance both livelihood-related and infrastructure-related public services?

This study is significant in both theoretical and practical terms. Theoretically, it fills an important gap by shifting the focus from urban-centric studies to the dynamics at the county level, where financial inclusion challenges are most acute. Drawing on Pigou’s (1920) [13] equalization theory, the research integrates welfare economics with spatial sociology perspectives (Lefebvre, 1991 [14]; Harvey, 1992 [15]; Soja, 2013 [16]) to provide new insights into how digital finance can reshape public service accessibility and promote spatial justice. Practically, the findings are expected to offer valuable guidance for policymakers by identifying targeted strategies for redistributing financial resources and improving public service delivery in underdeveloped county regions.

The primary objective of this research is to analyze both the direct and indirect effects of digital inclusive finance on public service quality at the county level. To achieve this, we construct a comprehensive evaluation framework that categorizes public services into livelihood-related and infrastructure-related sectors. Employing a suite of econometric techniques—including benchmark regression, mediation effect models, and threshold effect models—our analysis aims to rigorously quantify the mechanisms through which digital inclusive finance promotes service improvement. This methodological approach not only addresses the central research questions but also provides a robust empirical basis for understanding how digital finance can mitigate regional disparities.

In summary, by systematically investigating the interplay between digital inclusive finance and county-level public service provision, this study addresses a critical real-world problem and offers valuable theoretical and practical contributions. The analysis elucidates how digital inclusive finance not only serves as a catalyst for financial innovation and resource reallocation but also plays a vital role in enhancing public service quality. By clarifying the key challenges and underlying mechanisms influencing both digital financial services and public service delivery, the findings provide robust empirical evidence and actionable policy recommendations aimed at reducing regional disparities, improving service provision, and promoting sustainable socio-economic development in underdeveloped areas.

## 2. Literature review

### 2.1. Research on public services

The quality of public services is a key indicator of government governance capacity and social development (Lapuente and Van, 2020 [17]), encompassing various areas such as education, healthcare, ecological environment, municipal infrastructure, and transportation. Numerous studies have shown that the quality of public services is influenced not only by fiscal expenditure but also by factors such as regional economic development, residents’ savings levels, industrial structure, and policy support (Huang and Lan, 2025 [18]). Wang et al. (2024) [19] found that fiscal decentralization and local government competition play a crucial role in enhancing public service quality, particularly in underdeveloped regions, where the government’s resource allocation capacity directly affects the accessibility and equity of public services. In recent years, with the development of the digital economy, intelligent governance methods have gradually been applied in the public service sector, improving resource utilization efficiency and optimizing the supply of public services (Ejjami, 2024 [20]). However, research on county-level public services remains limited, with most studies focusing on provincial or municipal levels, neglecting the unique characteristics of counties as a key administrative unit.

### 2.2. Research on digital inclusive finance

As a key component of the digital economy, digital inclusive finance has played an active role in improving financial accessibility, reducing financing costs, and optimizing resource allocation (Fan and Chen, 2022 [21]). Jin and Liu (2024) [22] pointed out that the widespread adoption of digital financial technologies can effectively narrow the financial service gap and improve credit accessibility for low-income groups and small and medium-sized enterprises. Particularly in China, the government has vigorously promoted the development of digital inclusive finance, facilitating the rise of new financial models such as electronic payments, internet credit, and supply chain finance (Hasan et al., 2022 [6]). In recent years, scholars have gradually focused on the heterogeneous impact of digital inclusive finance across different regions and groups. For instance, Telukdarie and Mungar (2023) [23] found that digital finance is more effective in promoting economic growth in developed regions, while in underdeveloped areas, its primary role is to expand access to basic financial services. Although existing studies have explored the impact of digital inclusive finance on economic growth and residents’ well-being, systematic research on its effect on county-level public services remains limited.

### 2.3. Research on the impact mechanisms of digital inclusive finance on public services

In recent years, scholars have begun to focus on the impact mechanisms of digital inclusive finance on public services. Some studies suggest that digital finance can indirectly improve the quality of public services by enhancing local economic resilience (Yang et al., 2024 [24]), promoting urban innovation and industrial upgrading (Li and Li, 2022 [25]; Li et al., 2022 [26]), and improving the efficiency of financial resource allocation (Fan and Chen, 2022 [21]). Yang and Gong (2025) [27] found that digital inclusive finance significantly improves public service equity, particularly in regions with advanced innovation capabilities and mature technological infrastructure, revealing two key mechanisms: narrowing the urban-rural gap and optimizing industrial structure. Pazarbasioglu et al. (2020) [28] further pointed out that digital finance not only promotes individual consumption and entrepreneurship but also drives business credit growth, raising overall social income levels and thereby increasing the demand for public services. Other studies have shown that there is a synergy between digital inclusive finance and public service development, with digital inclusive finance enhancing the positive impact of public services on rural revitalization (Jiang, 2024 [29]). Additionally, some research has explored the impact of digital inclusive finance on specific public service sectors, such as education and healthcare (Tay et al., 2022 [30]), but systematic studies on its overall impact at the county level remain scarce.

### 2.4. Gaps in existing literature and extensions of this study

Despite existing research on public service quality, digital inclusive finance, and their interrelations, there are still several gaps: First, current studies are largely focused on macro levels, such as national or provincial levels, with limited attention paid to counties as key administrative units. Second, most studies concentrate on the impact of digital inclusive finance on economic growth and individual well-being, with little exploration of its specific effects on public service quality. Third, there is a lack of in-depth analysis on how digital inclusive finance influences different types of public services, such as livelihood-related public services and infrastructure-related public services. Additionally, existing research predominantly employs linear analytical methods, failing to fully reveal the nonlinear impacts of digital inclusive finance on public services.

To address these gaps, this article makes several contributions: First, it constructs a comprehensive evaluation system for county-level public service quality, covering five dimensions: education, healthcare, ecology, municipal services, and transportation. Second, based on data from 1445 counties, it employs threshold, mediation, and moderation effect models to systematically explore the impact pathways and nonlinear characteristics of digital inclusive finance on county-level public service quality. Third, it further analyzes the role of factors such as industrial structure and credit depth in this process, aiming to provide both theoretical and empirical support for policy formulation.

## 3. Theory and hypotheses

The rapid development of digital inclusive finance has profoundly impacted public services, yet the specific mechanisms of this influence remain systematically unexamined. To gain a deeper understanding of the complex mechanisms through which digital inclusive finance affects public services, this article will explore the multiple effects of digital inclusive finance across different dimensions and propose corresponding hypotheses.

### 3.1. Direct mechanisms of digital inclusive finance on public services

#### 3.1.1. Direct impact of digital inclusive finance on public services.

Digital inclusive finance plays a key role in improving the balance and supply of public services by enhancing financial infrastructure, which fosters the equalization of financial services. Through its financing function, it addresses long-term capital needs and boosts service supply efficiency (Ozili, 2018 [31]). Additionally, the big data-driven credit assessment capabilities of digital finance help manage risks in public service projects, improving service delivery effectiveness. Widespread use of digital and third-party payments also enhances residents’ access to services, driving up service consumption. Furthermore, digital finance platforms support the equalization of public information services by enabling functions like information queries (Kassen, 2022 [32]). However, its impact on public services depends on coverage, depth of use, and digitization; more extensive coverage, integration, and digitization correlate with improved service efficiency (Durai & Stella, 2019 [33]).

Despite its benefits, digital inclusive finance has potential drawbacks. The digital divide in underdeveloped areas may limit access to services, exacerbating inequalities (Roberts & Lunsford, 2023 [34]). Cybersecurity and privacy concerns also undermine trust in digital finance systems. The shift to digital finance might disrupt traditional services, causing job losses and reducing coverage. Moreover, information asymmetry may hinder informed decision-making, affecting equitable service distribution.

In conclusion, digital inclusive finance has both positive and negative effects on county-level public service quality. These mixed impacts underscore its complexity and the need for careful management. Thus, we propose Hypothesis 1: Digital inclusive finance significantly impacts the quality of county-level public services.

#### 3.1.2. Threshold effect of credit depth in digital inclusive finance.

The depth of credit assessment in digital inclusive finance is pivotal for evaluating the creditworthiness of individuals and institutions. Key elements include advanced risk assessment models, comprehensive credit information collection and sharing, and the generation of credit reports and scores. Personalized services, such as tailored loans and credit consulting, further enhance clients’ ability to manage credit effectively. Together, these components promote financial inclusion, risk control, innovation, and sustainability in the financial sector (Koul et al., 2024 [35]).

Insufficient credit depth, however, hampers financial institutions’ ability to accurately assess borrowers’ creditworthiness, raising loan thresholds and limiting access to financial resources. This restricts regional development and the improvement of public services. Incomplete borrower information leads to inaccurate assessments, resulting in higher interest rates, stricter loan conditions, and reduced capital access for businesses. These barriers hinder business growth, technological innovation, and investments in public services. Furthermore, stringent lending practices favor borrowers with strong credit histories, leaving those with weaker records unable to secure funding for essential development projects like infrastructure and public services.

In summary, inadequate credit depth within digital inclusive finance creates barriers that limit financial access, constraining the development and provision of public services. Therefore, Hypothesis 2 is proposed: The direct impact of digital inclusive finance on county-level public services depends on the depth of credit assessment.

### 3.2. Indirect mechanisms of digital inclusive finance on public services

#### 3.2.1. Mediating role of the tertiary industry.

Digital inclusive finance significantly enhances county-level public service quality by fostering the development of the tertiary industry and boosting employment within this sector. It achieves this primarily through loan support, enabling small and micro enterprises to access funding (Jin and Liu, 2024 [22]). These businesses, vital to county economies, use the funds to expand operations, diversify offerings, and improve services, which stimulate economic growth. This growth drives infrastructure development, including roads and public amenities, thereby improving public service quality.

Furthermore, digital inclusive finance creates employment opportunities in the labor-intensive tertiary sector. By promoting business growth, it generates jobs, raises income levels, and increases demand for public services. This heightened demand incentivizes governments to invest more in essential services such as healthcare, education, and social welfare. The increased need for skilled labor also encourages the development of education and training programs, further enhancing public service quality.

Additionally, financial product innovations introduced by digital inclusive finance—such as microloans and supply chain finance—leverage advanced technologies to meet the specific needs of tertiary businesses. These innovations improve financial service precision and efficiency, supporting industry growth, job creation, and an improved economic environment.

Digital inclusive finance also helps businesses expand markets and optimize marketing strategies through technology (Sudiantini et al., 2023 [36]). By opening new sales channels, businesses increase revenue, which can be reinvested into public services, such as upgrading schools and hospitals. This reinvestment strengthens public infrastructure and service coverage.

In conclusion, digital inclusive finance stimulates tertiary industry growth, fosters job creation, and enhances economic structures in county-level regions. These factors collectively improve the scope and quality of public services. Therefore, Hypothesis 3 is proposed: Digital inclusive finance influences county-level public service quality by promoting the development of the tertiary industry.

#### 3.2.2. Mediating role of financial loans.

Digital inclusive finance significantly improves the financial loan environment at the county level, thereby enhancing public service quality. It achieves this by increasing loan processing efficiency through digital platforms that streamline applications, approvals, and disbursements (Sudiantini et al., 2023 [36]). These advancements shorten business cycles, attract more loan applicants, and drive economic development, which indirectly improves public services.

Precise credit assessment systems are another key factor. Leveraging big data and artificial intelligence, digital inclusive finance reduces lending risks and allows higher credit limits. These larger loans enable investments in essential infrastructure, such as schools and hospitals, improving the reach and quality of public services.

Risk-sharing mechanisms between local governments, guarantee institutions, and financial organizations further encourage lending. By mitigating risks, these collaborations expand loan volumes, supporting local businesses and development projects, which directly benefit public services.

The diversification of financing channels, including the introduction of social capital, financial innovations, and international financing, supports technological advancements (Gomber et al., 2018 [37]). These resources enable the development of smart public services, such as modernized healthcare, education systems, and smart cities, increasing public service efficiency and accessibility.

Customized financial products tailored to local needs also play a vital role. Products like agricultural credit and microloans address specific economic demands, fostering industry growth and increasing local fiscal revenue. This revenue can be reinvested in infrastructure and public service improvements, strengthening county-level public service quality.

Lastly, improved service levels in financial institutions, achieved through training and capacity-building initiatives, enhance client satisfaction. This increase in competitiveness attracts more customers and boosts loan volumes, providing additional funding for public service investments.

In conclusion, digital inclusive finance improves the financial loan environment by enhancing efficiency, establishing robust credit systems, sharing risks, diversifying funding sources, offering tailored products, and raising service standards. These improvements create the financial foundation necessary for better infrastructure, education, healthcare, and social welfare. Hypothesis 4 is proposed: Digital inclusive finance affects county-level public service quality by optimizing the financial loan environment.

#### 3.2.3. Moderating effect of primary industry.

The impact of digital inclusive finance on public service quality is significantly moderated by the development of the primary industry, a critical economic component in many county-level regions of China. In areas where industrialization remains incomplete, sectors such as agriculture, forestry, animal husbandry, and fishery play a vital role in shaping economic and social outcomes, thereby influencing the relationship between digital inclusive finance and public service quality (Liu and Ren, 2023 [38]).

Counties with robust primary industries benefit from abundant natural and land resources, which provide essential infrastructure for digital finance development. Resources such as surplus land and water from agricultural activities enable infrastructural improvements that support financial accessibility and effectiveness, enhancing the reach and impact of digital inclusive finance.

Additionally, growth in the primary industry stimulates related sectors, fostering a more diversified industrial base. Digital inclusive finance supports this expansion by improving the efficiency of agricultural e-commerce, logistics, and distribution systems. These advancements reduce costs, enhance competitiveness, and create demand for complementary public services, such as logistics infrastructure and digital payment systems, ultimately improving public service quality.

In counties where the primary industry remains a significant GDP contributor, increased output can drive economic growth and expand fiscal resources. Prosperous counties are better equipped to invest in technological advancements, digital transformation, and infrastructure development. These investments not only bolster digital inclusive finance but also support public services by improving education, technical talent availability, and technological infrastructure.

Given these dynamics, it can be concluded that the primary industry’s development level plays a moderating role in the relationship between digital inclusive finance and public service quality. Hypothesis 5 is proposed: The level of primary industry development moderates the effect of digital inclusive finance on the quality of county-level public services.

#### 3.2.4. Moderating effect of fiscal revenue.

Higher local fiscal revenue significantly moderates the impact of digital inclusive finance on county-level public service quality by providing critical support for its development (Yan et al., 2023 [39]). This moderating effect is reflected in several key areas.

First, increased fiscal revenue enables local governments to invest in the expansion of digital inclusive finance through direct support to enterprises, digital infrastructure development, and the integration of digital technologies. These investments enhance the innovation and competitiveness of digital finance, improving the quality of public services.

Additionally, higher fiscal revenue facilitates talent cultivation and technological innovation. Governments can allocate resources to education and training programs in digital finance and related fields, fostering a skilled workforce that drives the growth of the digital finance sector. This growth indirectly contributes to improved public services through technological and professional advancements.

Moreover, optimizing fiscal revenue systems allows governments to implement targeted policies, such as tax incentives, subsidies, and support for digital finance enterprises. These measures attract businesses, promote the integration of digital finance with traditional industries, and stimulate regional economic growth. The resulting economic prosperity further enhances the quality of public services.

Based on these dynamics, it is hypothesized that county-level fiscal revenue moderates the effect of digital inclusive finance on public service quality. Hypothesis 6 is proposed: The level of county fiscal revenue moderates the effect of digital inclusive finance on the quality of county-level public services.

## 4 Materials and methods

### 4.1 Data source and data processing

This study focuses on 1445 counties in China, with the research scope defined as county-built-up areas to ensure data comparability and the reliability of research findings. Data selection is primarily based on the China County Statistical Yearbook and the China County Urban Construction Statistical Yearbook, including only counties recorded in both sources and prioritizing those with relatively complete data. To ensure data quality, a missing data exclusion criterion is set: counties with missing values exceeding 20% for key indicators are removed from the research sample. Based on data availability, this article selects 1445 counties across China from 2008 to 2022 as samples, comprising 21675 observations. The research conclusions can not only provide a detailed explanation of the situation in China but also serve as a reference for the study of other developing countries. Furthermore, it can provide empirical reference for policy-making in developing areas regarding the use of digital inclusive finance to improve the efficiency of public services in counties. This study is a quantitative analysis based solely on secondary data from economic and social statistical yearbooks and does not involve human participants; ethical approval was therefore not required.

The data for the dependent variable, public service quality evaluation index, and most control variables are primarily sourced from the Statistical Bulletin of the National Economic and Social Development, China County Statistical Yearbook, and China County Urban Construction Statistical Yearbook, which are published by the National Bureau of Statistics of China and the official websites of various county governments. Some data on ecological and environmental protection public services dimension are obtained from panel data on PM2.5 concentration quality in Chinese counties released by Washington University in St. Louis. The core explanatory variable, digital inclusive finance, mainly comes from the Peking University Digital Inclusive Finance Index (PKU-DFIIC) and the China County Digital Inclusive Finance Index Report, which contains data on the coverage breadth, depth of use, and digitalization level of digital inclusive finance in 31 provinces, 337 cities at and above the prefecture level, and nearly 2,800 counties in mainland China, providing support for research on digital inclusive finance. The remaining control variable data are sourced from the China Real Estate Statistical Yearbook, the Visible China Wind Night Light Database, EPS Database, and CEIC Database. This article adopts the Multiple Imputation (MI) method based on repeated simulation and performs data imputation using the “mice” package called in the R language programming.

To eliminate the impact of inflation, this article adopts the GDP deflator index with 2015 as the base year to adjust the economic indicators. Subsequently, all indicators are standardized. Finally, the entropy weighting method is employed for objective weighting to calculate the indicator weights, thereby deriving the comprehensive quality index of public services (ser ) and the digital inclusive finance index (dig) for each county in China over the years.

### 4.2 Variable selection

#### 4.2.1 Dependent variable.

In this article, the comprehensive quality index of public services (ser), derived from the indicator system, is set as the dependent variable. Following the principles of comprehensiveness, scientific rigor, continuity, comparability, and data accessibility, this article constructs a county-level public service evaluation system. The framework is based on the National Basic Public Service Standards (2023 Edition) and draws on the indicator selection methods of Li (2022) [40], Hui and Ning (2023) [41]. It encompasses five key dimensions—compulsory education, healthcare, social security, ecological environment protection, and municipal infrastructure and transportation—comprising 27 tertiary indicators. To eliminate differences caused by variations in population size across counties, all indicators in this article are measured using relative indicators, as shown in Table 1.

**Table 1:** Evaluation System for County-Level Public Service Quality.

Primary Indicator	Secondary Indicator	Tertiary Indicator	Unit	Indicator Trend	Weight of Secondary Indicator: Secondary Indicator/ Primary Indicator	Weight of Tertiary Indicator: Tertiary Indicator/ Secondary Indicator
County-Level Public Service	Education	Number of Ordinary Elementary School Students per 10,000 People	Persons/10,000 People	Positive	3.16%	60.53%
		Number of Ordinary Middle School Students per 10,000 People	Persons/10,000 People	Positive		39.47%
	Healthcare and Social Security	Number of Hospital Beds per 10,000 People	Beds/10,000 People	Positive	10.47%	22.18%
		Number of Beds in Various Social Welfare Institutions per 10,000 People	Beds/10,000 People	Positive		77.82%
	Ecological Environment	Average PM2.5 Concentration	Micrograms/Cubic Meter	Negative	17.56%	6.01%
		Carbon Emissions per 10,000 Yuan of GDP	Tons/10,000 Yuan	Negative		1.72%
		Sewage Treatment Rate	%	Positive		1.23%
		Per Capita Park and Green Space Area	Square Meters/Person	Positive		9.58%
		Green Space Ratio	%	Positive		4.15%
		Garbage Treatment Rate	%	Positive		1.18%
		Number of Sanitation Vehicles and Equipment per 10,000 People	Units/10,000 People	Positive		76.13%
	Municipal Infrastructure	Per Capita Daily Domestic Water Consumption	Liters/Person	Positive	46.29%	7.42%
		Proportion of Water Users to Total Households	%	Positive		6.98%
		Water Supply Coverage	%	Positive		1.12%
		Gas Supply Coverage	%	Positive		2.08%
		Sewer Pipe Density	Kilometers/Square Kilometer	Positive		8.22%
		Per Capita Fixed Asset Investment in Municipal Public Facilities	RMB/Person	Positive		19.09%
		Per Capita Comprehensive Water Supply Capacity	Cubic Meters/Day·Person	Positive		14.08%
		Length of Water Supply Pipelines per 10,000 People	Kilometers/10,000 People	Positive		14.31%
		Per Capita Water Supply for Public Services	Cubic Meters/Person	Positive		13.58%
		Number of Public Toilets per 10,000 People	Units/10,000 People	Positive		13.11%
	Transportation	Per Capita Road Area	Square Meters/Person	Positive	22.50%	15.29%
		Road Network Density	Kilometers/Square Kilometer	Positive		11.50%
		Length of Roads per 10,000 People	Kilometers/10,000 People	Positive		17.67%
		Number of Road Lighting Lamps per 10,000 People	Units/10,000 People	Positive		35.64%
		Proportion of Road Length with Installed Streetlights to Total Road Length	%	Positive		11.38%
		Proportion of Road Cleaning Area to Total Road Area	%	Positive		8.51%

Among them, education, medical care, and ecology are three dimensions used to measure the level of basic public service supply related to people’s livelihood. These three dimensions cover basic rights to survival, dignity, and capability, as well as basic health needs, and are closely related to people’s livelihoods. Municipal facilities and transportation facilities are two dimensions used to measure the level of infrastructure-related public service supply. These two dimensions cover the basic infrastructure needs for production and work, and are related to the infrastructure construction of the economy and society. Based on the constructed indicator system, this article calculates corresponding indices for the quality of public services related to people’s livelihoods (Livser) and infrastructure (Infser), and includes these indices as dependent variables in the theoretical model for analysis.

#### 4.2.2 Explanatory variables.

The explanatory variable is set as Digital Inclusive Finance (dig). This article adopts conventional research methods commonly used in academia, drawing from the approaches of Guo and Xiong (2021) [42] as well as Xie and Su (2021) [43]. The development level of digital inclusive finance is represented using the China Digital Inclusive Finance Index, released by the Digital Finance Research Center of Peking University. Its subsidiary indicators, namely “Coverage Breadth, Depth of Use, and Degree of Digitalization,” serve as robustness checks for the digital inclusive finance index.

#### 4.2.3 Threshold variable, mediator variables, and control variables.

The depth of credit provision in digital inclusive finance is set as the threshold variable (thres). The number of employees in the tertiary industry and the amount of loans provided by financial institutions are designated as mediator variables (media). Considering the possibility of other external environmental variables affecting public services, and to mitigate endogeneity issues, the following control variables are included in the analysis, as inspired by the studies of Zhang et al. (2021) [44], as well as Su and Li (2023) [45]. See Table 2 for details.

**Table 2:** Control Variables.

Criterion Level	Variable Level	Variable Symbol	Unit
Economic Development	Per Capita GDP	pGDP (per capita GDP)	yuan/person
Social Consumption	Per Capita Retail Sales of Consumer Goods	Consum (per capita commodity consumption)	yuan/person
Industrial Structure	Number of Secondary Industry Employees per Ten Thousand People	Sec (secondary industry employees per ten thousand people)	person/ten thousand people
	Number of Tertiary Industry Employees per Ten Thousand People	Ter (tertiary industry employees per ten thousand people)	person/ten thousand people
Financial Level	Per Capita Financial Loan Balance	Loan (per capita financial loan amount)	yuan/person
Population Scale	Population Density	Den (population density)	person/square kilometer
Construction Intensity	Construction Land Area per Ten Thousand People	Construc (construction land area per ten thousand people)	square kilometers/ten thousand people
Agricultural Level	Cultivated Land Area per Ten Thousand People	Culti (cultivated land area per ten thousand people)	hectares/ten thousand people
	Per Capita Output Value of Agriculture, Forestry, Animal Husbandry, and Fisheries	Pri (per capita primary industry output value)	yuan/person
Natural Endowment	Air Circulation Coefficient	Air (air flow coefficient)	/
Financial Level	Per Capita General Budgetary Revenue of Local Finance	Rev (per capita local fiscal revenue)	yuan/person

### 4.3 Model

#### 4.3.1 Static panel data model.

To examine the linear impact of digital inclusive finance on public services, the authors specify the following static double fixed-effects panel model:


$${ser}_{it}=\alpha +\beta_{1}{dig}_{it}+\beta_{2}{con}_{it}+\mu_{i}+\lambda_{t}+\varepsilon_{it}$$ (1)

Where i represents the county, t represents the year, $ser_{it}$ represents the quality of public services, $dig_{it}$ represents digital inclusive finance, $con_{it}$ represents the control variable group, α represents the intercept term, $\mu_{i}$ represents individual effects, $\lambda_{t}$ represents time effects, and $\varepsilon_{it}$ represents independently and identically distributed random disturbance terms.

To further examine the nonlinear impact of digital inclusive finance on public services, the square term of digital inclusive finance ($dig_{it}^{2}$) is introduced as an explanatory variable. The model is constructed as follows:


$${ser}_{it}=\alpha +\beta_{1}{dig}_{it}+\beta_{2}{dig}_{it}^{2}+\beta_{3}{con}_{it}+\mu_{i}+\lambda_{t}+\varepsilon_{it}$$ (2)

Model (2) focuses on examining the relationship between $\beta_{1}$ and $\beta_{2}$. When $\beta_{1}$ > 0 and $\beta_{2}$ < 0, it indicates an inverted “U-shaped” relationship between digital inclusive finance and public services. Conversely, when $\beta_{1}$ < 0 and $\beta_{2}$ > 0, it suggests a “U-shaped” relationship between digital inclusive finance and public services.

Building upon this, to test the moderating effect of digital inclusive finance on public services under macroeconomic and social variables, the following moderated effects panel model is constructed:


$${ser}_{it}=\alpha +\beta_{1}{dig}_{it}+\beta_{2}{mo}_{it}+\beta_{3}{dig}_{it}\times {mo}_{it}+\beta_{4}{con}_{it}+\mu_{i}+\lambda_{t}+\varepsilon_{it}$$ (3)

In Model (3), where $mo_{it}$ represents macroeconomic and social level moderating variables, and $dig_{it}\times mo_{it}$ represents the interaction term between digital inclusive finance and the moderating variables, other variables are explained as in Models (1) and (2). The focus of Model (3) is to observe the magnitudes of the coefficients of the core explanatory variable $\beta_{1}$ and the interaction term $\beta_{3}$. When $\beta_{1}$ > 0 and $\beta_{3}$ > 0, or $\beta_{1}$ < 0 and $\beta_{3}$ < 0, it indicates that the moderating variable strengthens the impact of digital inclusive finance on public services. Conversely, when $\beta_{1}$ > 0 and $\beta_{3}$ < 0, or $\beta_{1}$ < 0 and $\beta_{3}$ > 0, it suggests that the moderating variable weakens the impact of digital inclusive finance on public services.

#### 4.3.2. Panel threshold model.

For examining whether there exists a nonlinear heterogeneous impact of digital inclusive finance on public services, this article adopts Hansen’s (2017) [46] panel threshold model, with the following basic formula:


$$\begin{array}{c} {ser}_{it}=\alpha +\eta_{1}{dig}_{it}\times I\left({thres}_{it}\leq v_{1}\right)+\eta_{2}{dig}_{it}\times I\left(v_{1}<{thres}_{it}\leq v_{2}\right)\\ +\cdots +\eta_{m}{dig}_{it}\times I\left(v_{m-1}<{thres}_{it}\right)+\beta {con}_{it}+\mu_{i}+\lambda_{t}+\varepsilon_{it} \end{array}$$ (4)

Wherein, $thres_{it}$ represents the threshold variable, I(·) is an indicator function taking values of 1 or 0. It equals 1 if the condition inside the parentheses is satisfied, and 0 otherwise. $v_{1}$ to $v_{m-1}$ are the threshold values to be estimated, dividing the sample into multiple intervals. The coefficients $\eta_{1}$ to $\eta_{m}$ of digital inclusive finance levels differ across different intervals.

#### 4.3.3. Mediation effect model.

To examine the indirect effect mechanism of digital inclusive finance on public services, the authors adopt the three-stage mediation model proposed by Wen et al. (2022) [47]. Building upon Model (1), the authors establish the mediation effect model and employ two methods—stepwise regression and Bootstrap test—to verify the mediation mechanism. The mediation effect model is set as follows:


$${ser}_{it}=\alpha_{0}+\alpha_{1}{dig}_{it}+\alpha_{2}{con}_{it}+\mu_{i}+\lambda_{t}+\varepsilon_{it}$$ (5)

$${media}_{it}=\beta_{0}+\beta_{1}{dig}_{it}+\beta_{2}{con}_{it}+\mu_{i}+\lambda_{t}+\varepsilon_{it}$$ (6)

$${ser}_{it}=\theta_{0}+\theta_{1}{dig}_{it}+\theta_{2}{media}_{it}+\theta_{3}{con}_{it}+\mu_{i}+\lambda_{t}+\varepsilon_{it}$$ (7)

Where: $media_{it}$ represents the mediator variable. The regression focuses on the size and direction of coefficients $\alpha_{1}$, $\beta_{1}$, $\theta_{1}$, and $\theta_{2}$.

The main steps are as follows: First, test the total effect $\alpha_{1}$ of digital inclusive finance on the basic public service level in Model (5). If $\alpha_{1}$ is significant, proceed to the next step. Secondly, examine whether the impact efficiency $\beta_{1}$ of digital inclusive finance on the mediator variable is significant in Model (6). If significant, continue the test. Finally, if $\theta_{2}$ in Model (7) is significant, it indicates the presence of a mediation effect. If both $\theta_{1}$ and $\theta_{2}$ are significant and $\theta_{1}$ <$\alpha_{1}$, it implies a partial mediation effect. If $\theta_{1}$ is not significant, it indicates a complete mediation effect. It’s important to note that using the mediation effect model to address economic issues should be based on economic theory rather than simple statistical logic.

## 5. Results

### 5.1. Baseline regression results

Before conducting the baseline regression, a series of tests were conducted to demonstrate the rationality of the model and variable selection. Firstly, to avoid multicollinearity among explanatory variables, a multicollinearity test was performed. The results showed that the maximum variance inflation factor (VIF) was 5.03, with an average of 2.00, indicating that the VIF values were within an acceptable range, thus ruling out the problem of multicollinearity. Secondly, the F-test was conducted, and the regression results showed significant P-values, suggesting that the fixed effects or random effects models were more suitable than the pooled effects model. The Hausman test showed significant P-values, indicating the selection of the fixed effects model. Ultimately, the bidirectional fixed effects model, which eliminates heteroscedasticity, was chosen to examine the relationship between digital inclusive finance and the comprehensive quality of public services, quality of public services related to people’s livelihood, and quality of infrastructure-related public services, as shown in Table 3. Columns (2), (5), and (8) include the control variables. To further investigate the presence of nonlinear relationships, columns (3), (6), and (9) include the squared term of the explanatory variable digital inclusive finance.

**Table 3:** Baseline Regression Results.

Explanatory Variables and Control Variables	Dependent Variable								
	ser			Livser			Infser		
	Column (1)	Column (2)	Column (3)	Column (4)	Column (5)	Column (6)	Column (7)	Column (8)	Column (9)
dig	−0.0079129* (−2.21)	−0.0071452* (−2.10)	−0.1417232*** (−4.19)	0.18683*** (3.79)	0.2271466** (3.25)	0.4622188*** (8.37)	−0.0065836** (−2.48)	−0.0075989** (−2.68)	−0.1459804*** (−6.07)
pGDP		−0.0026317* (−1.90)	−0.0023904 (−1.51)		−0.4510458*** (−3.84)	−0.4513513*** (−3.85)		0.0399419** (2.59)	0.0396497** (2.64)
Consum		0.0454388*** (5.89)	0.0384071*** (6.48)		0.0439887** (3.09)	0.0623458*** (3.54)		0.0309906*** (4.34)	0.0233748*** (4.13)
Sec		0.0004588 (0.34)	0.0002383 (0.19)		−0.0742788** (−2.92)	−0.0712811** (−2.77)		−0.0099639* (−1.91)	−0.0099755* (−2.02)
Ter		0.0097022*** (3.68)	0.0100931*** (3.63)		0.0240641 (1.51)	0.0234023 (1.47)		0.0093177*** (4.77)	0.0096245*** (4.73)
Loan		0.06663*** (6.99)	0.0705762*** (7.93)		−0.0228033 (−1.18)	−0.0236974 (−1.28)		0.0618921*** (3.76)	0.065406*** (4.22)
Den		−0.0163059 (−1.68)	−0.0146242 (−1.39)		−0.0266505 (−1.58)	−0.0278418 (−1.77)		−0.0077513 (−0.67)	−0.0067716 (−0.57)
Construc		−0.0063815** (−3.40)	−0.0052215** (−2.72)		−0.0244177 (−1.67)	−0.0266136 (−1.73)		−0.0036526 (−1.82)	−0.0025156 (−1.17)
Culti		−0.0062377 (−0.20)	−0.0031339 (−0.09)		−0.8099895*** (−6.09)	−0.8245315*** (−6.11)		0.0017638 (0.67)	0.0031375 (1.12)
Pri		−0.003663*** (−4.83)	−0.0042299*** (−4.57)		0.0320088 (0.47)	0.0392693 (0.57)		−0.0041412*** (−5.87)	−0.004829*** (−7.65)
Air		0.0351189* (1.95)	0.0354174* (2.06)		−0.0569981 (−0.58)	−0.0598383 (−0.59)		0.035438** (2.56)	0.0365091** (3.19)
Rev		0.0003332 (0.15)	0.0001042 (0.05)		0.0326492 (1.58)	0.0330806 (1.62)		−0.0006448 (−0.72)	−0.0007809 (−0.85)
dig^2^			0.1374521*** (5.07)			−0.2965214** (−3.02)			0.1481291*** (6.93)
Intercept term	0.2049871*** (51.42)	0.1741287*** (20.76)	0.2146639*** (14.92)	−2.910908*** (−165.83)	−2.712374*** (−18.99)	−2.766037*** (−21.28)	0.1444243*** (48.92)	0.1198863*** (21.43)	0.1640826*** (15.13)
Fixed individual effects	Yes	Yes	Yes	Yes	Yes	Yes	Yes	Yes	Yes
Fixed time effects	Yes	Yes	Yes	Yes	Yes	Yes	Yes	Yes	Yes
F-test	140.05***	76.92***	73.78***	86.28***	59.57***	59.70***	87.60 ***	49.36 ***	46.08***
Hausman test	26.41***	48.03***	50.50***	19.01***	55.29***	59.12***	26.81***	38.67***	43.19***
Sample size	21675	21675	21675	21675	21675	21675	21675	21675	21675
R–squared	0.6325	0.6601	0.6678	0.6195	0.6614	0.6626	0.4518	0.4957	0.5102

Note: *, **, *** represent significance at the 10%, 5%, and 1% levels respectively. The values in parentheses are the corresponding t-statistics.

Columns (1) and (2) in Table 3 respectively present the fixed effects analysis results of digital inclusive finance on the comprehensive quality of public services before and after adding control variables. It is observed that regardless of whether control variables are included, the regression coefficient of digital inclusive finance is negative at the 10% significance level, indicating a significant impact of digital inclusive finance on the comprehensive quality of public services. In column (2), after controlling for other variables, the coefficient of digital inclusive finance is −0.0071452, which is significant at the 10% significance level. This suggests that for each percentage point increase in digital inclusive finance, the comprehensive quality of public services in counties will decrease by 0.7 percentage points. Considering the diversity of public services in counties, the development of digital inclusive finance may have heterogeneous effects on different types of public service quality, hence the need for classification tests.

Observing columns (4) and (5), it is found that digital inclusive finance has a significant positive impact on the quality of public services related to people’s livelihood, with a coefficient of 0.2271466, significant at the 5% significance level after controlling for other variables. By observing columns (7) and (8), it is found that digital inclusive finance has a significant negative impact on the quality of infrastructure-related public services. In summary, there is an asymmetrical impact of digital inclusive finance on public service quality of different categories, and there is still significant room for improvement in supporting infrastructure-related public services. The reasons for this could be that digital inclusive finance tends to focus more on short-term returns and liquidity, usually preferring to invest in areas that can quickly generate higher returns with lower risks, such as education and healthcare, while infrastructure-related public services typically take longer to break even and start generating profits, and usually require substantial investment with higher associated risks. Additionally, infrastructure projects often require government leadership, and the market mechanisms may not be mature enough, leading to a lack of interest or confidence among private investors, thus affecting the quality of infrastructure-related public services.

In terms of control variables, per capita consumption expenditure has a significant positive effect on the quality of public services related to people’s livelihood and infrastructure-related public services. A higher per capita consumption expenditure usually implies more spending, which can promote the improvement of public services related to people’s livelihood because the government can obtain more funds from consumption taxes and other channels to enhance services such as education and healthcare. Similarly, a higher consumption expenditure also facilitates the improvement of infrastructure-related public services as it generates more tax revenue, which can be allocated to infrastructure construction and maintenance. Per capita GDP has a significant negative impact on the quality of public services related to people’s livelihood and a significant positive impact on the quality of infrastructure-related public services. The differing effects are attributed to the fact that higher per capita GDP may indicate higher living standards and purchasing power, enabling residents to afford advanced public services available in the market, thereby reducing the demand for basic public services provided by the government. However, it also provides more funds for improving infrastructure-related public services. The number of employees in the tertiary industry, financial loans, and air circulation have significant positive effects on the quality of infrastructure-related public services, but their effects on the quality of public services related to people’s livelihood are not significant. The increase in the number of employees in the tertiary industry is usually associated with the development of the service industry and economic growth, which requires higher quality infrastructure to support more economic activities, thereby promoting the improvement of infrastructure-related public services. An increase in financial loans can provide more social mobility funds for investment and improvement of infrastructure-related public services. Improved air circulation contributes to the maintenance and prolongation of the service life of infrastructure. However, population density, construction land area, and local fiscal revenue do not have a significant impact on both types of public service quality.

To further examine nonlinear relationships, refer to columns (3), (6), and (9) in Table 3. Column (3) shows that the coefficient of digital inclusive finance on the comprehensive quality of public services is −0.1417232, significant at the 1% level. The coefficient of the squared term of digital inclusive finance on the comprehensive quality of public services is 0.1374521, significant at the 1% level, indicating a “U”-shaped left-half curve relationship between digital inclusive finance and the comprehensive quality of public services, whereby the negative impact of digital inclusive finance on the comprehensive quality of public services gradually diminishes as the level of digital inclusive finance increases. Comparing columns (6) and (9), the impact of digital inclusive finance on the quality of public services related to people’s livelihood exhibits an inverted “U”-shaped left-half curve relationship, while its impact on the quality of infrastructure-related public services shows a “U”-shaped left-half curve relationship. The reason may be that in the field of public services related to people’s livelihood, the initial increase in the level of digital inclusive finance may bring substantial investment, but as the investment increases, there may be issues such as improper resource allocation or declining investment efficiency, leading to a decreasing marginal impact on the quality of public services related to people’s livelihood. Investment in infrastructure-related public services has a long cycle and slow returns. The funds introduced through digital inclusive finance in the early stages may not be fully utilized, leading to low investment efficiency and consequently affecting the quality of infrastructure-related public services. As the level of digital inclusive finance further increases, related regulatory mechanisms may be strengthened, and the efficiency of fund utilization may be optimized, thereby reducing the risk of overinvestment or resource wastage.

It is worth noting that the impact of digital inclusive finance on public service quality is not static. It tends to exhibit different effects in response to changes in the external environment. As the primary industries in most counties in China still revolve around the agricultural sector, the interaction term between digital inclusive finance and the output value of the primary industry is included in the baseline regression model to examine the moderating effect of the primary industry’s output value on the relationship between digital inclusive finance and public service quality. The results are presented in Table 4.

**Table 4:** Moderating Effect Regression Results.

The explanatory variables	ser	
	Column (1)	Column (2)
dig	−0.0070416* (−1.96)	−0.0065115* (−1.96)
Pri	−0.0045633*** (−7.80)	−0.0046853** (−3.22)
dig × Pri	0.002082* (2.09)	0.0025076** (2.50)
Control variables	No	Yes
Intercept term	0.1988682*** (45.94)	0.1777701*** (16.85)
Fixed individual effects	Yes	Yes
Fixed time effects	Yes	Yes
Sample size	21675	21675
R–squared	0.6348	0.6393

Note: *, **, *** represent significance at the 10%, 5%, and 1% levels respectively. The values in parentheses are the corresponding t-statistics.

Columns (1) and (2) show that the coefficient of the interaction term between digital inclusive finance and the output value of the primary industry is significantly positive. This indicates that the development of the primary industry in counties mitigates the negative impact of digital inclusive finance on public service quality. It underscores the vital intervention role of the primary industry in the influence of digital inclusive finance on public service quality in counties. Therefore, encouraging counties to consolidate and sustain the development of the primary industry is crucial. The primary industry plays a significant role in the county economy, providing employment opportunities and supporting stable economic development. Measures such as strengthening agricultural modernization, optimizing the agricultural industry structure, enhancing infrastructure construction, supporting technological innovation, and expanding financial services can further increase the output value of the primary industry. Consequently, this can alleviate the adverse effects of digital inclusive finance on public service quality and promote comprehensive development in county economies.

### 5.2. Threshold model regression results

In this article, the credit depth of digital inclusive finance is designated as the threshold variable to examine the threshold effect of digital inclusive finance on public service quality at different levels of credit depth. The threshold effect test primarily involves two steps: firstly, examining the existence and number of thresholds, and secondly, conducting significance tests for the identified threshold values. To determine the threshold values, the bootstrap method is employed, iterated 1000 times, to calculate the significance of the threshold test and the threshold critical value of the credit depth of digital inclusive finance.

From the results of Table 5, the F-test value passes the single threshold test at the 1% level, while the double and triple threshold tests are not significant, indicating the presence of a single threshold in the impact of digital inclusive finance on public service quality. The corresponding threshold value, measured by the minimum residual sum of squares, is 0.6629. These results suggest the existence of a threshold effect in the impact of digital inclusive finance on public service quality. Further estimation of the threshold effect is conducted.

**Table 5:** Regression Results of Panel Threshold Model.

Variable	Coefficient	Standard Error	t-value	p-value
dig < 0.6629	−0.0433212*	0.0247745	−1.75	0.085
dig > 0.6629	0.2068522***	0.0729462	2.84	0.006
pGDP	−0.0031301	0.0045952	−0.68	0.498
Consum	0.0312071	0.0228361	1.37	0.176
Sec	0.0005438	0.0032239	0.17	0.867
Ter	0.0102722***	0.0031683	3.24	0.002
Loan	0.0819929***	0.0306697	2.67	0.009
Den	−0.0148055	0.0206531	−0.72	0.476
Construc	−0.0043566	0.0077691	−0.56	0.577
Culti	−0.0037497	0.0321881	−0.12	0.908
Pri	−0.0040933	0.0030256	−1.35	0.180
Air	0.0440298*	0.023925	1.84	0.070
Rev	−0.0000535	0.0026941	−0.02	0.984
Intercept	0.1963576***	0.024354	8.06	0.000
Fixed Effects	Yes			
Time Effects	Yes			
Sample Size	21675			
R-squared	0.6853			
Threshold Value	0.6629			
Single Threshold Test – p	0.0033			
Double Threshold Test – p	0.6800			
Triple Threshold Test – p	0.6433			

Note: *, **, *** represent significance at the 10%, 5%, and 1% levels, respectively.

Looking at the regression results of the single threshold model, the coefficient of digital inclusive finance on public service quality shows variation across two intervals, exhibiting an overall “U”-shaped trend, which is consistent with the non-linear test results of the squared term in the baseline regression as discussed earlier. When the depth of credit is below 0.6629, digital inclusive finance has a significantly negative impact on public service quality. However, once the depth of credit surpasses the threshold value of 0.6629, the impact of digital inclusive finance on public service quality turns positive, with a coefficient of 0.2068522, significant at the 1% level. Moreover, the absolute values of the corresponding t-values increase as the depth of credit rises, indicating that increasing the depth of credit enhances the empowering effect of digital inclusive finance on public service quality.

The reason behind this phenomenon lies in the fact that digital inclusive finance in most counties of China is still in its early stages of development, resulting in relatively low levels of credit depth. Under such circumstances, issues like the imperfect financial credit evaluation system make it difficult for local enterprises and residents to access sufficient financial support, thereby constraining their ability to improve living standards and access public services, consequently impeding the development of public services. However, once the depth of credit in digital inclusive finance is enhanced, it will contribute to improving the credit evaluation systems of financial institutions for local enterprises and residents, making financial institutions more willing to provide more and better financial services to local clients. Through this mechanism, local economies will receive more support, and social public services will be improved, thereby promoting the economic development and social progress of the counties.

### 5.3. Indirect effect test

In order to further explore whether the development level of the tertiary industry and the amount of loans from financial institutions play an indirect role in the impact of digital inclusive finance on the quality of public services, this article draws on relevant research by Wen et al. (2022) [47], Jiang (2022) [48], to examine the influence of core explanatory variable (dig) on intermediary variables(media).

Model (1) in Table 6 presents the test results using the number of employees in the tertiary industry as a mediating variable. In Model (1), Column (1) shows that the coefficient $\alpha_{1}$ of digital inclusive finance (DIF) is −0.0071452, significant at the 10% level, indicating a significant impact of DIF on public service quality. In Column (2), the coefficient value $\beta_{1}$ of DIF on the mediator variable is 0.0759616, significant at the 1% level, suggesting that the development of DIF significantly enhances the level of third-industry development in the county. In Column (3), the coefficient value $\theta_{2}$ is 0.0097022, significant at the 1% level, indicating that the improvement in the level of third industry development significantly promotes public service quality. Bootstrap test was conducted with 1000 repeated samples, and the results showed that the confidence intervals for the mechanism of action of third industry development did not include 0, indicating a significant mediating effect of third industry development. Model (1) demonstrates that DIF can significantly enhance the development level of the third industry, which in turn strengthens the quality of public services in the county. Therefore, this article suggests that DIF can empower public service quality by improving the development level of the third industry.

**Table 6:** Mediation Effect Regression Results.

Variables	Model (1): Development of the tertiary industry			Model (2): Amount of loans from financial institutions		
	Column(1)Path α_1_ ser	Column(2)Path β_1_ Ter	Column(3)Path θ_2_ and θ_1_ ser	Column(4)Path α_2_ ser	Column(5)Path β_2_ Loan	Column(6)Path θ_4_ and θ_3_ ser
dig	−0.0071452* (−2.10)	0.0759616*** (3.71)	−0.0071452* (−2.10)	−0.0071452* (−2.10)	0.0126695*** (4.41)	−0.0071452* (−2.10)
Ter			0.0097022*** (3.68)			
Loan						0.06663*** (6.99)
Intercept	0.1741287*** (20.76)	−0.6994825** (−3.12)	0.1741287*** (20.76)	0.1741287*** (20.76)	0.1298204*** (6.98)	0.1741287*** (20.76)
Control variables	Yes	Yes	Yes	Yes	Yes	Yes
Fixed individual effects	Yes	Yes	Yes	Yes	Yes	Yes
Fixed time effects	Yes	Yes	Yes	Yes	Yes	Yes
Sample size	21675	21675	21675	21675	21675	21675
R–squared	0.6601	0.4159	0.6601	0.6601	0.2173	0.6601
Bootstrap Z	2.37			1.98		
Bootstrap Z–p	0.018			0.047		

Note: *, **, *** indicate significance at the 10%, 5%, and 1% levels respectively, with the corresponding t-values in parentheses.

In addition, digital inclusive finance (DIF) can also enhance the quality of public services in the county by increasing the loan amount of financial institutions. Model (2) presents the test results using the loan amount of financial institutions as a mediating variable. It can be observed that the coefficient $\beta_{2}$ in Model (2) is 0.1298204, significant at the 1% level, indicating that DIF significantly increases the loan amount of financial institutions. Furthermore, the coefficient $\theta_{4}$ is 0.06663, significant at the 1% level, suggesting that the higher the loan amount of financial institutions, the higher the quality of public services in the county. Bootstrap test indicates significance, hence this article concludes that the loan amount of financial institutions also exhibits a significant mediating effect.

The above results indicate that digital inclusive finance (DIF) can enhance the quality of public services by promoting the development of the county’s tertiary industry and increasing the loan amount of financial institutions. The development of the tertiary industry stimulates economic activities and employment opportunities, improves residents’ living standards, increases fiscal revenue, and fosters innovation. At the same time, the increase in the loan amount of financial institutions provides more financial support to enterprises and individuals in the county, driving infrastructure construction and improvements in public services. Therefore, this article concludes that enhancing the development of the tertiary industry and increasing the loan amount of financial institutions are crucial channels through which digital inclusive finance promotes the high-quality development of public services.

### 5.4. Endogeneity test

To mitigate endogeneity concerns, the authors first conducted a Hausman test and a heteroscedasticity-robust Durbin-Wu-Hausman (DWH) test on the two-stage least squares regression (2SLS). The results indicated that digital inclusive finance could be considered an endogenous variable.

Endogeneity issues can arise from three main sources, including measurement errors, omitted variables, and reverse causality. The variable selection and index construction in this article have largely minimized endogeneity resulting from measurement errors and omitted variables. To address potential reverse causality, the authors followed the approach of Zhao et al. (2020) [49], Yang et al. (2023) [50], using the interaction term of the “Digital Inclusive Finance Index” and the “Number of Microblogging Enterprises per Ten Thousand People in 2022” as instrumental variable (iv) for digital inclusive finance. The instrumental variable was included in the baseline model for regression analysis.

The choice of the instrumental variable is based on the integral relationship between digital inclusive finance and the internet. The internet serves as a crucial channel for economic entities to access information, and the development of digital inclusive finance is closely intertwined with internet growth. Platforms like Weibo, as typical social media platforms, are core products of internet technology and can effectively reflect whether regional development is closely integrated with internet growth. While controlling for a series of variables, the influence of the instrumental variable on public service quality is limited, satisfying the relevance and exogeneity conditions of instrumental variables.

The weak instrument test results in Table 7 indicate that the Cragg-Donald Wald F statistic exceeds the 10% critical value provided by Stock-Yogo, suggesting the absence of weak instrument problems. Furthermore, the identification test’s Kleibergen-Paap rk LM statistic rejects the null hypothesis at the 10% level, demonstrating the instrument’s identifiability and thus indicating the rationality and effectiveness of the selected instrument variables.

**Table 7:** The estimation results using the Heckman two-step method.

	Column (1)	Column (2)	Column (3)	Column (4)
	The First Stage	The Second Stage	The First Stage	The Second Stage
Variable	dig	ser	dig	ser
iv	−0.3102968*** (−4.31)		−0.3142288*** (−4.33)	
dig		−0.0150366** (−2.04)		−0.0117887* (−1.75)
Control Variables	No	No	Yes	Yes
Fixed Effects	Yes	Yes	Yes	Yes
Time Effects	Yes	Yes	Yes	Yes
F-test	18.57***		18.72***	
Identification Test	2.96*		3.00*	
Weak Instrument Test	1079.59 (16.38)		1061.49 (16.38)	
Hansen J	0.000		0.000	
Observations	21675		21675	
R-squared	0.6304		0.6593	

Note: Weak instrument test employs the Cragg-Donald Wald F-statistic (critical value at 10% level in parentheses). Identification test uses the Kleibergen-Paap rk LM statistic (p-value reported in parentheses).

Introducing the instrument variables into the baseline regression model, the regression estimation of the model is conducted using two-stage least squares (2SLS), with the results reported in Table 7. In the first-stage regression results (Column 1), the coefficient of the instrument variables is significant at the 1% level, indicating a significant correlation between the instrument variables and digital inclusive finance. In the second-stage regression results (Column 2), the impact of digital inclusive finance on public service quality remains significant, consistent with the previous baseline regression findings. This suggests that after considering endogeneity issues, the results of the baseline regression model are somewhat robust, indicating that endogeneity has not influenced the main conclusions of this article.

### 5.5. Robustness checks

To ensure the robustness of the regression results, this article employs the following methods for examination. (i) Using the “breadth of digital inclusive finance coverage” indicator as a substitute for the original core explanatory variable, the comprehensive “digital inclusive finance” index, to conduct regression analysis. (ii) Regression Using Lagged Core Explanatory Variables: Considering the lagged effect of digital inclusive finance on public service quality, regression analysis is conducted using the core explanatory variable lagged by one period. (iii) Winsorization to Remove Outliers: To mitigate the influence of outliers from specific time periods and individual counties, winsorization is applied to the panel data with a two-sided trimming of 1% before regression. Regardless of the method employed, the estimation results of the core explanatory variable remain highly consistent with the baseline regression results, demonstrating the robustness of the baseline regression results.

## 6. Conclusion

### 6.1. Main conclusion

(i) Baseline regression results show that digital inclusive finance has heterogeneous effects on public services: it reduces overall service quality but enhances livelihood-related services while weakening infrastructure services. This disparity arises from digital finance’s focus on short-term, high-yield sectors like education and healthcare, rather than long-term, high-risk infrastructure projects. Nonlinear analysis reveals a U-shaped relationship with overall public service quality, an inverted U-shape for livelihood services, and a U-shape for infrastructure, suggesting that higher digital finance levels can optimize resource allocation. Additionally, a stronger primary industry mitigates digital finance’s negative impact on public services, emphasizing the need to enhance agricultural modernization, industry structures, and financial services to stabilize county economies and improve public services.
(ii) The threshold model results identify a single threshold effect of digital inclusive finance on public service quality, with a critical credit depth value of 0.6629. Below this threshold, digital finance negatively affects service quality, but once credit depth exceeds 0.6629, the effect becomes significantly positive, indicating a U-shaped relationship. This shift occurs because initial low credit depth limits financial access, hindering improvements in public services. As credit depth increases, enhanced financial mechanisms support local enterprises and residents, boosting economic development and service quality. The findings underscore the importance of deepening credit access to unlock digital finance’s positive impact on county economies.
(iii) The indirect effect test reveals that digital inclusive finance enhances public service quality through two main channels: tertiary industry development and increased financial institution loans. Digital inclusive finance boosts the tertiary industry, which improves service quality. Similarly, digital inclusive finance raises loan amounts from financial institutions, positively impacting service quality. These findings underscore the importance of strengthening the tertiary industry and expanding financial support as key mechanisms for improving public service quality in county economies.

### 6.2. Research value

The conclusions of this study directly address the research questions posed in the introduction, particularly regarding the direct and indirect effects of digital inclusive finance on public service quality at the county level. The study identifies both positive and negative impacts of digital finance on public services, highlighting the mechanisms—such as credit depth, tertiary industry development, and increased financial loans—that explain these effects. These findings are consistent with the research questions by demonstrating how digital inclusive finance influences public services through financial reallocation, industrial upgrading, and improved financial access. Thus, the conclusions effectively correspond to the guiding questions, providing valuable insights for policy-making aimed at improving public service provision.

This study provides a novel perspective on the relationship between digital inclusive finance and public service quality, contributing to the literature on digital economy and public service equity. By integrating welfare economics and spatial equity theory, the research extends existing knowledge by highlighting how digital finance influences both civilian-oriented and infrastructure-related services. It reveals that digital inclusive finance can improve civilian welfare but also suggests the need for optimized resource allocation to strengthen infrastructure services. These findings align with Pigou’s welfare economics theory, which emphasizes inequality in resource distribution and advocates for targeted interventions to achieve social welfare (Pigou, 1920 [13]). Additionally, the study uncovers a threshold effect in digital finance’s impact on public service quality, consistent with nonlinear economic convergence theory. It shows that regions can unlock the positive potential of digital finance once a critical credit depth is surpassed, resonating with the inverted U-shaped development curve in convergence theory (Azariadis and Drazen, 1990 [51]). Furthermore, this research highlights the moderating role of primary industry output, contributing to spatial sociology theory by illustrating the interaction between local industry and public service provision (Soja, 1989 [52]). This suggests that financial strategies should be tailored to regional economic structures.

This study also provides significant practical contributions by offering guidance for policymaking, especially in the context of China. The identification of the threshold effect in digital finance penetration suggests that ensuring sufficient credit depth is critical for unlocking the positive impacts of digital finance on public service quality. Furthermore, the study underscores the importance of designing region-specific strategies, considering the moderating influence of the primary industry, which emphasizes the need for policies that align digital finance development with local economic conditions. The research also highlights the roles of tertiary industry development and increased financial institution loans in improving public services. Strengthening these areas can further enhance public service outcomes, offering valuable insights for optimizing digital finance’s contribution to county economies. This also aligns with endogenous growth theory (Romer, 1986 [53]), suggesting that digital finance, when combined with industry and financial development, can drive continuous improvements in public service quality.

### 6.3. Policy recommendations

Considering the aforementioned conclusions, the following recommendations are proposed:

(i) Enhancing Digital Inclusive Finance Credit Depth: Governments should focus on increasing the credit depth of digital inclusive finance to mitigate its negative effects and boost county-level public service development. This can be achieved by expanding digital finance applications and improving financial resource allocation efficiency, particularly in civilian-oriented public services like education and healthcare. Additionally, reforms should encourage financial institutions to innovate digital finance products and services tailored to county-level areas, providing practical support for public service improvement.
(ii) Accounting for External Environmental Factors: Governments should enhance the primary industry in counties while promoting the development of the tertiary industry. This creates a supportive environment for digital inclusive finance to positively impact public services. Efforts should include fostering cross-regional cooperation to enhance the flow of technology, talent, and capital, and facilitating income growth through industrial advancement. Additionally, promoting digital transformation of traditional industries and strengthening the financial institution loan environment will further amplify the benefits of digital inclusive finance in the public sector.
(iii) Integrating Digital Inclusive Finance with Public Services: Differentiated policies should be implemented to integrate digital inclusive finance with public services, especially in counties with lower public service quality and less developed digital finance infrastructure. Governments should support the digitization and intelligent transformation of public services and encourage collaboration between internet finance companies and public service construction. Expanding the coverage of digital inclusive finance in public services will enhance overall service quality.

This study has deeply explored the impact of digital inclusive finance on public service quality at the county level in China, but it still has limitations. Future research could integrate big data and artificial intelligence techniques to improve analytical accuracy. Additionally, it could be expanded to different countries and regions, especially those with varying economic development levels, to enhance the generalizability of the findings. Moreover, future studies should consider incorporating policy environments and cultural factors into the analysis to uncover more complex mechanisms of influence.

## Supporting information

S1 Data Raw dataset used in the analysis. (XLSX)

## References

[1] Wang Y, Chi Y. Measuring Spatiotemporal changes of rural basic public service in poverty-stricken area of China. Int Regional Sci Rev. 2016;41(5):510–39. doi: 10.1177/0160017616665671

[2] Wang S, Zhang Q, Sun M, Teng Y. Has urban public service equalization reduced regional differences in economic resilience?. PLoS One. 2024;19(8):e0303236. doi: 10.1371/journal.pone.0303236 39186741 PMC11346925

[3] Jia D, Cheng C. Research on the imbalanced dilemma and strategies of teacher resources in county-level compulsory education in China: based on grounded theory. J Hebei University Sci Technology (Social Science Edition). 2025;25(1):47–54.

[4] Wang Z, Wang X. Research on the imbalance in bed allocation of county-level adoption-based social welfare institutions. Journal of Northeastern University (Social Science Edition). 2023;25(1):74–87. doi: 10.15936/j.cnki.1008-3758.2023.01.009

[5] Chen B, Zhao C. Poverty reduction in rural China: Does the digital finance matter?. PLoS One. 2021;16(12):e0261214. doi: 10.1371/journal.pone.0261214 34914740 PMC8675699

[6] Hasan MdM, Yajuan L, Khan S. Promoting China’s Inclusive Finance Through Digital Financial Services. Global Business Review. 2020;23(4):984–1006. doi: 10.1177/0972150919895348

[7] Kanungo RP, Gupta S. Financial inclusion through digitalisation of services for well-being. Technological Forecasting Social Change. 2021;167:120721. doi: 10.1016/j.techfore.2021.120721

[8] Ozili PK. Financial inclusion research around the world: A review. Forum for Social Econ. 2020;50(4):457–79. doi: 10.1080/07360932.2020.1715238

[9] Mhlanga D. Blockchain for digital financial inclusion towards reduced inequalities. FinTech and Artificial Intelligence for Sustainable Development: The Role of Smart Technologies in Achieving Development Goals. Cham: Springer Nature Switzerland. 2023. p. 263–90.

[10] Tran HTT, Le HTT. The Impact of Financial Inclusion on Poverty Reduction. Asian J Law Econ. 2021;12(1):95–119. doi: 10.1515/ajle-2020-0055

[11] Martinez Gutiérrez C, Krauss A. What drives financial inclusion at the bottom of the pyramid? Empirical evidence from microfinance panel data. 2015.

[12] GGnther MK. The Progress of Financial Inclusion in India: Insights from Multiple Waves of Survey Data. SSRN J. 2017. doi: 10.2139/ssrn.2946954

[13] Pigou AC. The Economics of Welfare. 4th ed. London: Macmillan. 1920.

[14] The production of space. Choice Reviews Online. 1992;30(03):30-1449-30–1449. doi: 10.5860/choice.30-1449

[15] HARVEY D. Social Justice, Postmodernism and the City*. Int J Urban Regional Res. 1992;16(4):588–601. doi: 10.1111/j.1468-2427.1992.tb00198.x

[16] Seeking spatial justice. Choice Reviews Online. 2011;48(05):48-3000-48–3000. doi: 10.5860/choice.48-3000

[17] Lapuente V, Van de Walle S. The effects of new public management on the quality of public services. Governance. 2020;33(3):461–75. doi: 10.1111/gove.12502

[18] Huang X, Lan F. The influencing factors and regional heterogeneity of county-level public services quality in China. Int J Urban Sciences. 2025;:1–33. doi: 10.1080/12265934.2025.2452502

[19] Wang Y, Huang X, Zhang T, Jiang B, Wang X. Impact of fiscal decentralization and local government competition on the supply of basic public services: Based on the empirical evidence of prefecture-level cities in China. Heliyon. 2024;10(4):e26511. doi: 10.1016/j.heliyon.2024.e26511 38420436 PMC10901029

[20] -RE. Public Administration 5.0: Enhancing Governance and Public Services with Smart Technologies. IJFMR. 2024;6(4). doi: 10.36948/ijfmr.2024.v06i04.26086

[21] Fan Y, Chen ST. Research on the effects of digital inclusive finance on the efficiency of financial resource allocation. Front Environ Sci. 2022;10. doi: 10.3389/fenvs.2022.957941

[22] Jin L, Liu M. Unlocking Financial Opportunities: The Substantial Alleviation of Financing Constraints on Small and Micro Enterprises Through Digital Inclusive Finance. J Knowl Econ. 2024;16(1):2283–309. doi: 10.1007/s13132-024-01863-7

[23] Telukdarie A, Mungar A. The Impact of Digital Financial Technology on Accelerating Financial Inclusion in Developing Economies. Procedia Computer Sci. 2023;217:670–8. doi: 10.1016/j.procs.2022.12.263

[24] Yang Y, Lin Z, Xu Z, Liu S. The impact of digital finance on regional economic resilience. Pacific-Basin Finance Journal. 2024;85:102353. doi: 10.1016/j.pacfin.2024.102353

[25] Li J, Li B. Digital inclusive finance and urban innovation: Evidence from China. Rev Development Econ. 2021;26(2):1010–34. doi: 10.1111/rode.12846

[26] Li F, Wu Y, Liu J, Zhong S. Does digital inclusive finance promote industrial transformation? New evidence from 115 resource-based cities in China. PLoS One. 2022;17(8):e0273680. doi: 10.1371/journal.pone.0273680 36037191 PMC9423615

[27] Yang Y, Gong B. Digital financial inclusion and public service equalization. Finance Res Lett. 2025;71:106440. doi: 10.1016/j.frl.2024.106440

[28] Pazarbasioglu C, Mora AG, Uttamchandani M, Natarajan H, Feyen E, Saal M. World Bank. 2020;54(1).

[29] Jiang Y. Public service equalization, digital financial inclusion and the rural revitalization: Evidence from Chinese 283 prefecture-level cities. International Rev Economics Finance. 2024;96:103648. doi: 10.1016/j.iref.2024.103648

[30] Tay L-Y, Tai H-T, Tan G-S. Digital financial inclusion: A gateway to sustainable development. Heliyon. 2022;8(6):e09766. doi: 10.1016/j.heliyon.2022.e09766 35785228 PMC9240988

[31] Ozili PK. Impact of digital finance on financial inclusion and stability. Borsa Istanbul Review. 2018;18(4):329–40. doi: 10.1016/j.bir.2017.12.003

[32] Kassen M. Blockchain and e-government innovation: Automation of public information processes. Information Syst. 2022;103:101862. doi: 10.1016/j.is.2021.101862

[33] Durai T, Stella G. Digital finance and its impact on financial inclusion. J Emerging Technologies Innovative Res. 2019;6(1):122–7.

[34] Roberts JA, Lunsford R. Reducing the financial inclusion gap through digital transformation. 2023.

[35] Koul S, Verma R, KV A. Privacy Preferences of Consumer and Lender: A Case of Digital Credit Systems in India. J Internet Commerce. 2024;23(4):414–44. doi: 10.1080/15332861.2024.2418175

[36] Sudiantini D, Rizky PP, Hazarika A. Digital economy and financial inclusion in reviving the national economy: A Management Strategy. RJME. 2023;1(1). doi: 10.61650/rjme.v1i1.245

[37] Gomber P, Kauffman RJ, Parker C, Weber BW. On the Fintech Revolution: Interpreting the Forces of Innovation, Disruption, and Transformation in Financial Services. J Management Information Syst. 2018;35(1):220–65. doi: 10.1080/07421222.2018.1440766

[38] Liu J, Ren Y. Can digital inclusive finance ensure food security while achieving low-carbon transformation in agricultural development? Evidence from China. J Cleaner Prod. 2023;418:138016. doi: 10.1016/j.jclepro.2023.138016

[39] Yan J, Tu X, Zheng J. Does digital economy strengthen the income distribution effect of fiscal expenditure? Evidence from China. PLoS One. 2023;18(8):e0290041. doi: 10.1371/journal.pone.0290041 37561747 PMC10414578

[40] Li B. Measurement of basic public service equalization level and regional differences in the Yangtze River midstream urban agglomeration. Statistics and Decision Making. 2022;38(1):53–8. doi: 10.13546/j.cnki.tjyjc.2022.01.011

[41] Hui N, Ning N. Study on the effect and mechanism of digital economy driving the improvement of public service quality. Journal of Beijing University of Technology (Social Sciences Edition). 2023;23(1):109–24.

[42] Guo F, Xiong Y. Measurement and impact study of digital inclusive finance in China: A literature review. Financial Rev. 2021;13(6):12–23, 117–8.

[43] Xie D, Su B. Digital inclusive finance supports rural revitalization development: theoretical analysis and empirical test. Shandong Social Sciences. 2021;(4):121–7. doi: 10.14112/j.cnki.37-1053/c.2021.04.019

[44] Zhang D, Guo H, Lu Y. The impact of fiscal transparency on the equalization of basic public services. Macroeconomic Research. 2021;(11):5–16. doi: 10.16304/j.cnki.11-3952/f.2021.11.001

[45] Su C, Li Z. Does the digital economy enhance government public service capacity? Modern Economic Discussion. 2023;(1):1–14.doi: 10.13891/j.cnki.mer.2023.01.011

[46] Hansen BE. Regression kink with an unknown threshold. J Business Economic Statistics. 2017;35(2):228–40.

[47] Wen ZL, Fang J, Xie JY. Methodological research on domestic mediation effects. Advances in Psychological Science. 2022;30(8):1692–702.

[48] Jiang T. Mediation and moderation effects in empirical research on causal inference. China Industrial Economy. 2022;(5):100–20. doi: 10.19581/j.cnki.ciejournal.2022.05.005

[49] Zhao T, Zhang Z, Liang S. Digital economy, entrepreneurship activity, and high-quality development: empirical evidence from Chinese cities. Management World. 2020;36(10):65–76. doi: 10.19744/j.cnki.11-1235/f.2020.0154

[50] Yang G, Wang H, Fan H. Carbon emission reduction effects of digital economy: theoretical analysis and empirical evidence. China Industrial Economy. 2023;(5):80–98. doi: 10.19581/j.cnki.ciejournal.2023.05.005

[51] Azariadis C, Drazen A. Threshold Externalities in Economic Development. The Quarterly Journal of Economics. 1990;105(2):501. doi: 10.2307/2937797

[52] Soja E. Postmodern geographies: The reassertion of space in critical social theory. London: Verso. 1989.

[53] Romer PM. Increasing Returns and Long-Run Growth. J Political Economy. 1986;94(5):1002–37. doi: 10.1086/261420

